# High-Magnification Object Tracking with Ultra-Fast View Adjustment and Continuous Autofocus Based on Dynamic-Range Focal Sweep

**DOI:** 10.3390/s24124019

**Published:** 2024-06-20

**Authors:** Tianyi Zhang, Kohei Shimasaki, Idaku Ishii, Akio Namiki

**Affiliations:** 1 Namiki Laboratory, Graduate School of Engineering, Chiba University, 1-33 Yayoi-cho, Inage-ku, Chiba-shi 263-8522, Chiba, Japan; namiki@faculty.chiba-u.jp; 2 Smart Robotics Laboratory, Graduate School of Advanced Science and Engineering, Hiroshima University, 1-4-1 Kagamiyama, Higashi-Hiroshima 739-8527, Hiroshima, Japan; simasaki@hiroshima-u.ac.jp (K.S.); iishii@robotics.hiroshima-u.ac.jp (I.I.)

**Keywords:** high-speed vision, object tracking, autofocus, liquid lens

## Abstract

Active vision systems (AVSs) have been widely used to obtain high-resolution images of objects of interest. However, tracking small objects in high-magnification scenes is challenging due to shallow depth of field (DoF) and narrow field of view (FoV). To address this, we introduce a novel high-speed AVS with a continuous autofocus (C-AF) approach based on dynamic-range focal sweep and a high-frame-rate (HFR) frame-by-frame tracking pipeline. Our AVS leverages an ultra-fast pan-tilt mechanism based on a Galvano mirror, enabling high-frequency view direction adjustment. Specifically, the proposed C-AF approach uses a 500 fps high-speed camera and a focus-tunable liquid lens operating at a sine wave, providing a 50 Hz focal sweep around the object’s optimal focus. During each focal sweep, 10 images with varying focuses are captured, and the one with the highest focus value is selected, resulting in a stable output of well-focused images at 50 fps. Simultaneously, the object’s depth is measured using the depth-from-focus (DFF) technique, allowing dynamic adjustment of the focal sweep range. Importantly, because the remaining images are only slightly less focused, all 500 fps images can be utilized for object tracking. The proposed tracking pipeline combines deep-learning-based object detection, K-means color clustering, and HFR tracking based on color filtering, achieving 500 fps frame-by-frame tracking. Experimental results demonstrate the effectiveness of the proposed C-AF approach and the advanced capabilities of the high-speed AVS for magnified object tracking.

## 1. Introduction

Object tracking is a crucial task in various fields such as robotics [1,2], autonomous driving [3,4], and video surveillance [5,6]. However, tracking small objects remains challenging due to their subtle appearance and limited distinguishing features [7].

In recent years, various methods have enhanced small object tracking. For instance, computer vision techniques [8,9] and high-definition (HD) video methods [10,11] have improved the ability to acquire small objects’ features. However, these methods have increased computational costs and data transmission loads. On the other hand, active vision systems (AVS) have been developed to obtain high-resolution regions of interest (ROI). To capture sufficiently rich details, high-magnification lenses are necessary. However, in high-magnification scenes, significant challenges of pan-tilt and focus adjustment arise due to the constraints of narrow field of view (FoV) and shallow depth of field (DoF) [12,13]. Specifically, the narrow FoV in high-magnification images necessitates that the AVS possesses high-speed pan-tilt adjustment capabilities. Without this, objects may move out of the field of view, resulting in a loss of tracking. Furthermore, the shallow DoF implies that objects can easily become defocused and undetectable if the autofocus (AF) mechanism employed is not sufficiently fast.

To enhance the pan-tilt adjustment speed, several studies have proposed utilizing mirror-based pan-tilt mechanisms [14,15]. Unlike conventional pan-tilt methods, which move the entire camera body, mirror-based methods adjust the viewpoint without moving the camera, significantly improving the control speed of the angle of view. However, AF approaches have not seen comparable progress.

In general, AF approaches are categorized into passive and active methods, with passive methods including contrast-based and phase-based passive AF methods [16,17]. Contrast-based methods determine the adjustment direction by comparing the sharpness of the current image to consecutive frames, necessitating multiple iterations to achieve optimal focus [18,19]. Phase-based methods utilize focus sensors to measure the phase difference of light, thereby reducing the number of iterations but sacrificing accuracy [20,21]. For continuous AF (C-AF) tasks, passive AF methods experience significant delays and result in unstable frame rates for focused images. In contrast, active AF directly measures object distance using additional sensors, which significantly speeds up the AF process [22,23]. However, the limited resolution and working range of depth sensors result in low precision when measuring small objects.

In contrast, to achieve stable frame rates of focused images, researchers have investigated methods based on focal sweep [24,25]. This technique moves the focusing lens group back and forth to capture multiple images at varying focus positions. Subsequently, focus measure algorithms are applied to select the sharpest image, resulting in the production of focused output [26,27]. However, this approach tends to yield an excessively low frame rate for tracking small, fast-moving objects, primarily due to the capture of numerous redundant images.

In this study, we present a high-speed AVS capable of swiftly tracking small, fast-moving objects and consistently capturing high-resolution well-focused, high-magnification images. The success of our approach in meeting this demanding task can be attributed to two key properties of the system:

- **C-AF Method Based on Dynamic-Range Focal Sweep:** We introduce a novel C-AF method that utilizes a 500 fps high-speed camera in conjunction with a liquid lens for dynamic-range focal sweep at 50 Hz. The optimal focused image is extracted using the focus measure method based on the Canny sharpness detector [28]. The adjustment of the focal sweep range is achieved by measuring the depth of the object through the Depth-from-Focus (DFF) technique [29,30]. This results in a narrowed focal sweep range around the object’s true focus, leading to stable 50 fps well-focused images. Moreover, due to the effective shortening of the focal sweep range, the blur in the remaining images, which are less focused, is minimal. Thus, all 500 fps images can be utilized for object tracking.
- **500 fps Frame-by-Frame High-Magnification Tracking Pipeline:** We propose a 500 fps frame-by-frame tracking method specifically designed for high-speed high-magnification tracking. The proposed HFR tracking pipeline utilizes the main color of the object, continuously updated by applying the K-means clustering method [31] on the ROI obtained by YOLOv8 [32]. Leveraging the ultra-fast pan-tilt mechanism based on the Galvano mirror, our system achieves 500 Hz view direction adjustment.

The subsequent sections of this paper are organized as follows. Section 2 discusses related works on AF approaches, conventional methods for object tracking, and the development of AVS for high-speed pan-tilt adjustment. Section 3 provides a detailed explanation of the key contributions of our work, including the proposed C-AF approach based on dynamic-range focal sweep and an HFR frame-by-frame object tracking pipeline. Section 4 describes the hardware utilized to form the proposed system and the recording of the computational time costs of each implemented algorithm. Section 5 presents the experiments conducted to demonstrate the advanced capabilities of the proposed C-AF approach and to showcase the advanced performance of high-magnification object tracking. Finally, conclusions are provided in Section 6.

## 2. Related Works

### 2.1. Development of Autofocus

AF approaches have been widely implemented in cameras to capture well-focused images [16,17]. Active AF employs depth sensors to directly measure object distances [22,23,33,34]. With active AF, focus adjustment can be completed in a single step. However, it faces challenges with small objects due to potential inaccuracies in distance measurement arising from limitations in sensor resolution and signal attenuation. Passive AF methods include phase-based and contrast-based approaches. Phase-based passive AF relies on focus sensors to detect the focus state [20,35,36]. This approach distinguishes adjustment directions by examining the phase gap of different lights separated by the micro-lens of the focus sensor. However, it cannot achieve non-delay C-AF because the re-focusing process starts only when the current phase state is determined to be out of focus. Contrast-based passive AF utilizes focus search methods to progressively adjust focus [37]. Although predictive algorithms such as depth from defocus [38,39] and machine learning [21,40,41] can reduce the number of iterative steps compared to traditional search methods like rule-based search [42] and fast climbing search [43], they cannot provide stable frame-rate focused images due to uncertain time costs in the refocusing process.

Recently, faster AF opto-mechanisms, including piezoelectric actuators [44], voice coil motors [45], and focus-tunable liquid lenses [46], have been developed, reducing focus adjustment times to mere tens of milliseconds per step. Building upon this, some studies have generated focused images across a wide and constant depth range using focal sweep techniques [24,25,47]. Moreover, our previous work has achieved DoF extension image generation at 15 fps by combining the global-range focal sweep with the multi-focus fusion technique based on high-speed vision [48]. However, considering that C-AF is typically applied for a single object, C-AF based on global-range focal sweep produces vast redundancy of frames, leading to a lower output frame rate [26,27].

Therefore, this paper introduces the C-AF approach based on dynamic-range focal sweep to achieve well-focused high-magnification images with a stable and sufficient frame rate. Moreover, because the focal sweep range is short, the blur in the less focused images is slight, allowing all images to be utilized for object tracking. In our work, with the proposed HFR object tracking pipeline, we achieve 500 fps frame-by-frame high-magnification tracking. A comparison of the proposed C-AF approach with previous methods is presented in Table 1.

### 2.2. Development of Object Tracking Algorithms

Object tracking is a fundamental task in computer vision, encompassing a diverse array of techniques to address its challenges and complexities. Generally, object tracking algorithms can be categorized into three main types: feature-based tracking, region-based tracking, and deep learning-based tracking. Feature-based methods extract object features, such as edge information [49], color information [50], histogram of oriented gradients (HoG) [51], and Haar-like features [52], which distinguish the object from the background regions. Region-based methods, such as the Kernelized Correlation Filter (KCF) tracker [53] and the Minimum Output Sum of Squared Error (MOSSE) tracker [54], track objects by identifying regions in subsequent frames that correlate with the ROI in the previous frame. Additionally, methods like MeanShift [55] and CamShift [56] can be hybridized with the aforementioned algorithms to improve tracking efficiency by adjusting tracking windows to regions with high feature density. However, due to variations in object features and correlations, these methods often fail in long-term tracking tasks due to their low robustness to environmental changes.

Recently, methods based on convolutional neural networks (CNNs) have significantly enhanced object detection capabilities, greatly improving robust long-term object tracking. Several studies have successfully tracked specific objects such as defects [57] and vehicles [58] using CNN-based methods. Moreover, advancements in parallel computation hardware have effectively accelerated the computation speed of CNNs [59], making deep-learning-based tracking methods feasible for real-time tracking. However, due to the high computational demands of such algorithms, applying them to HFR real-time tracking remains challenging.

To achieve real-time HFR image processing, high-speed vision techniques accelerate image processing algorithms to ensure that the algorithms implemented can be operated on images captured at dachshunds or even thousands of frames per second in real time [60]. This technique has been applied in various scenarios, including vibration testing [61], industrial automation [62], and video surveillance [63]. High-speed vision is particularly crucial for achieving high-frequency visual feedback, further essential for high-magnification tracking where high-speed pan-tilt adjustment is needed. For example, Jiang et al. introduced a 500 fps frame-by-frame object tracking system combining CNN-based object detection and KCF-based tracking [15]. However, the template of the KCF tracker often struggles to handle situations with rapid changes in object size. Although some improved methods have been proposed, they do not run sufficiently fast for HFR tracking [64,65].

In this study, we propose a hybrid tracking pipeline that combines deep-learning-based tracking and feature-based tracking. What distinguishes our method is its specific design for high-magnification scenes. Despite the high demand for high-frequency visual feedback in such scenes, the simplicity of object and background colors in high-magnification images allows us to fully utilize the color information of the object consistently updated by the object detection results based on deep learning.

### 2.3. Development of High-Speed Active Vision Systems

To obtain high-resolution images of specific objects, AVSs have been widely applied. AVSs utilize pan-tilt mechanisms to adjust the view directions horizontally and vertically, and through tracking algorithms, these systems can continuously obtain high-resolution images of moving objects. Cai et al. proposed an AVS to track people and capture high-magnified face images; however, this system is not applicable indoors due to its lack of AF mechanism [66]. Liu et al. proposed an eye gaze tracking system using an AVS with AF mechanism, achieving indoor small-object high-magnification tracking. Nevertheless, users are restricted to sitting in front of the system and cannot move quickly because the pan-tilt adjustment speed is insufficient for objects’ motions in high-magnification scenes [67].

In order to achieve high-speed AVSs, Galvano mirror-based ultra-fast pan-tilt adjustment methods have been proposed [68,69]. This approach departs from traditional active vision systems, which directly move the camera. Instead, it employs a two-axis Galvano motor to control the view direction within 2 ms. In recent years, Galvano mirror-based AVSs have been developed across various applications. This method has been introduced into microscopic imaging for view expansion and switching, greatly improving the FoV of microscopic systems [70,71]. In addition, Hu et al. developed a new depth measurement method by proposing a dual-view mechanism using a mirror reflection mechanism based on the Galvano mirror system [72]. Their team also designed a multiple body key-part tracking system with only one camera [73]. Using it as a base, they further expanded the FoV by combining it with a high-speed rotating mirror [74]. Recently, Li et al. enhanced this system to track multiple types of small objects simultaneously [75]. However, the fixed-focus lenses in these systems constrain their working depth range.

In contrast, our work combines high-speed C-AF and pan-tilt adjustment, achieving magnified object tracking across not only a wide FoV but also a deep depth range. A comparison of our system and previous AVSs is shown in Table 2.

## 3. High-Speed Continuous Autofocus High-Magnification Object Tracking

### 3.1. Overview of the Proposed System

**Figure 1:**
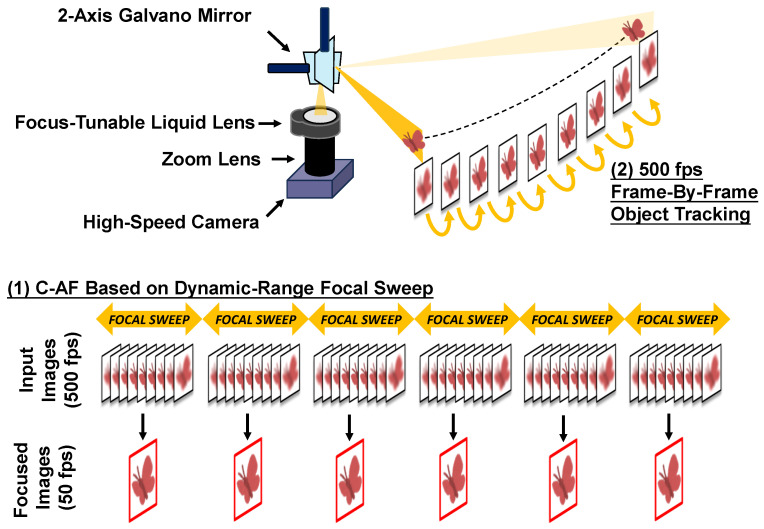
Overview of the proposed high-magnification object tracking system: The combination of (1) C-AF based on dynamic-range focal sweep and (2) 500 fps frame-by-frame object tracking achieves high-speed and precise high-magnification tracking of small objects moving in a wide scene.

As depicted in Figure 1, the proposed system encompasses two core functions: (1) a C-AF approach based on dynamic-range focal sweep, and (2) a 500 fps frame-by-frame object tracking pipeline. The integration of these functions effectively addresses the challenges posed by shallow DoF and narrow FoV, achieving advanced high-speed high-magnification object tracking.

Function (1) utilizes a high-speed camera operating at 500 fps and a focus-tunable liquid lens operating a sine wave at 25 Hz, resulting in focal sweeps around the object’s optimal focus and capturing 10 frames per sweep. A focus measure algorithm selects the best-focused image from these 10 frames as the output. Our C-AF method can output 50 fps stable focused images with 25 Hz focus range adjustment, effectively mitigating the challenge of shallow DoF in high-magnification scenes. Consequently, it enables the proposed system to accurately measure the object’s depth. Importantly, unlike the methods based on global-range focal sweep, the short focal sweep range ensures minimal blur in the less-focused images, thereby allowing all 500 fps images to be used for object tracking with Function (2).

Function (2) combines high-accuracy, long-term object detection based on YOLO with efficient object tracking based on the object’s main color, achieving 500 fps frame-by-frame tracking performance. An ultra-fast pan-tilt mechanism using a Galvano mirror enables precise pan-tilt adjustments at 500 Hz, successfully addressing the challenge of narrow FoV in high-magnification scenes.

The details of our methods are presented in the following subsections: Section 3.2 elucidates the technical details of our proposed C-AF approach. Section 3.3 introduces the optical geometry of the ultra-fast pan-tilt mechanism based on the Galvano mirror. Section 3.4 elaborates on our 500 fps frame-by-frame tracking pipeline, completing the objective of high-speed C-AF high-magnification object tracking.

### 3.2. Continuous Autofocus Based on Dynamic-Range Focal Sweep

#### 3.2.1. Optical Geometry of Focal Sweep

**Figure 2:**
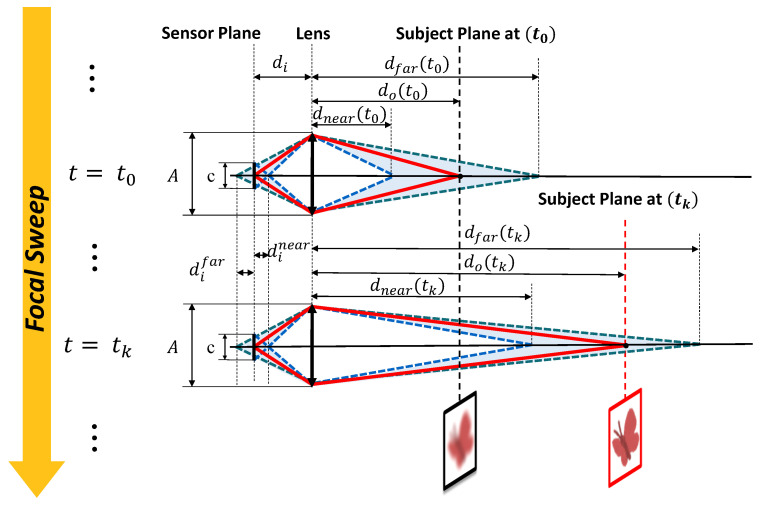
From time $t_{0}$ to $t_{k}$, the focus of the camera system changes with the variation of the diopter of the liquid lens. During each period, the high-speed camera captures multiple images with different focuses. Using the focus measure algorithm, the image with the best focus can be extracted. Simultaneously, through the correlation between the focal length and the distance of the subject plane, the object’s depth can be calculated.

Because of high focal sweep frequency, it can be assumed that the change in the object’s size and position during the focal sweep is negligible. Thus, the optical model can be simplified into the geometry based on the thin lens law, as described in Figure 2.

According to the Gaussian lens equation, the relationship between the focal length of the camera system *f*, the distance between the sensor plane and the lens $d_{i}$, and the distance between the subject plane at the lens $d_{o}$ can be expressed as Equation (1),

(1) $$\frac{1}{f}=\frac{1}{d_{o}}+\frac{1}{d_{i}},$$

where the focal length *f* is known through the equation $\frac{1}{f}=\frac{1}{f_{z}}+\frac{1}{f_{l}}$, where $f_{z}$ and $f_{l}$ represent the focal lengths of the zoom lens and the liquid lens, respectively.

According to the intercept theorem, the nearest and farthest distances between the lens and the sensor plane to capture focused images, denoted as $d_{i}^{near}$ and $d_{i}^{far}$, can be expressed in Equation (2),

(2) $$d_{i}^{near}=\frac{d_{i}}{1-\frac{c}{A}},\;d_{i}^{far}=\frac{d_{i}}{1+\frac{c}{A}},$$

where *A* refers to the aperture diameter, and *c* refers to the acceptable circle of confusion (CoC).

From Equations (1) and (2), given a specific focus position $d_{o}$, the nearest and the farthest distance of the DoF, defined as s $d_{near}$ and $d_{far}$, can be calculated by Equation (3),

(3) $$d_{near}=\frac{Afd_{o}}{Af+c(d_{o}-f)},\;d_{far}=\frac{Afd_{o}}{Af-c(d_{o}-f)}.$$

Therefore, to guarantee that there is at least one focused image captured during the focal sweep, the adjacent images’ gap should adhere to Equation (4),

(4) $$d_{near}\left(t_{k+1}\right)\leq d_{far}\left(t_{k}\right),$$

where the t(k) and t(k+1) refer to the capturing time of the first image and its subsequent image.

#### 3.2.2. Dynamic-Range Focal Sweep

**Figure 3:**
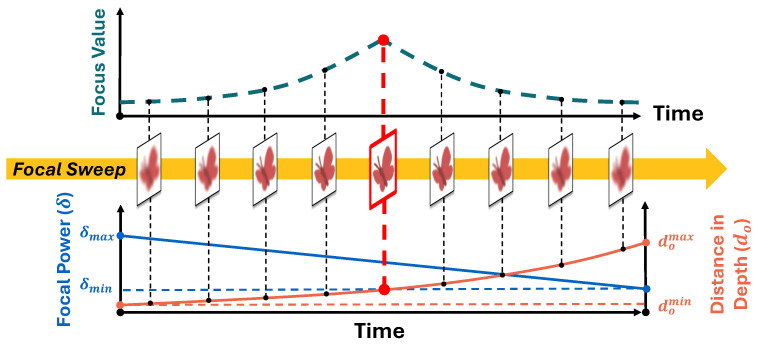
Schematic of depth measurement with focal sweep.

In our work, we utilize the GPU-accelerated Canny sharpness detector [28] on each image to obtain the focus value in real time. After each focal sweep, the image with the highest focus value is extracted. Meanwhile, by recording the capturing time, the distance of the object can be determined, as described in Figure 3.

The range of focal sweeps is controlled by setting the liquid lens’ diopter, i.e., focal power δ ($\delta =\frac{1}{f_{l}}$). We send the commands of the maximal and the minimal focal powers $\delta^{max}$ and $\delta^{min}$ while keeping the focal sweep’s frequency at 25 Hz. When the liquid lens is operated in the sinewave mode, we simplify the variation of δ as uniform due to its high-frequency movement. Therefore, in the forward focal sweeps, the focal power at a random time $t_{k}$ can be calculated by Equation (5),

(5) $$\delta \left(t_{k}\right)=\delta_{max}-\frac{\delta_{max}-\delta_{min}}{T}t_{k}\;\left(0\leq t_{k}\leq T\right),$$

where *T* is the period of the focal sweep.

Thus, in a forward focal sweep with maximal and minimal positions $d_{o}^{max}$ and $d_{o}^{min}$, the focus position of the image is expressed with Equation (6),

(6) $$d_{o}\left(t\right)=\frac{d_{o}^{max}d_{o}^{min}T}{\left(d_{o}^{min}-d_{o}^{max}\right)t+d_{o}^{max}T}\;\left(0\leq t\leq T\right),$$

where *t* is the capturing time of this image.

Therefore, by substituting the capturing time of the best-focused image, which can be determined using the focus measure algorithm, into the above equation, we can measure the depth of the object.

In our system, we manually define multiple focal sweep ranges with corresponding acceptable position intervals. The setting of the focal sweep ranges depends on the total focal length and the aperture of the lens. Additionally, the setup of the acceptable CoC also impacts the setting of the focal sweep ranges.

**Figure 4:**
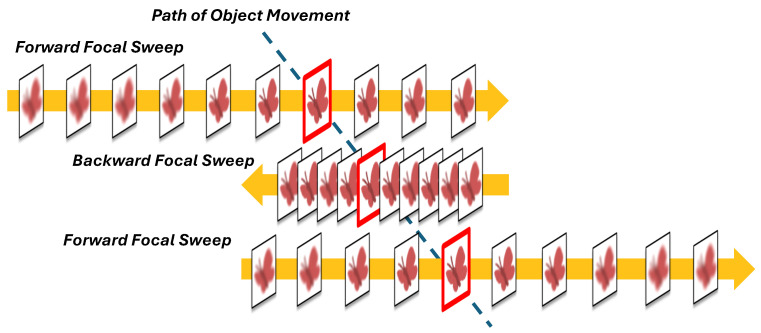
Diagram of the adjustment of the dynamic-range focal sweep: The first forward focal sweep measures the object’s depth. Then, at the backward focal sweep, the range of the focal sweep can be adjusted. At the second forward focal sweep, the system can finish the range adjustment and measure the object’s depth again.

The proposed mechanism of the dynamic-range focal sweep allows for adjusting the focal sweep to keep the object within the central interval when its distance exceeds the acceptable range. The diagram of this operation is shown in Figure 4.

Because the focal sweep range of the backward focal sweeps is not stable, we only use the forward focal sweeps to measure the object’s depth. In our work, the depth measurement frequency is at 25 Hz. While, the image with the highest focus value in both the forward focal sweeps and the backward focal sweeps is output, resulting in a 50 Hz well-focused image output. The details of the focus measure algorithm are introduced in Section 3.4.2, and the computational time cost per frame is recorded in Section 4.

The proposed C-AF approach based on dynamic-range focal sweep effectively reduces input image redundancy. With our system, it ensures stable 50 fps well-focused images, and all 500 fps images are focused enough to be utilized for high-magnification object tracking. To demonstrate its performance, Experiments 1, 2, and 3 are conducted.

### 3.3. Optical Geometry of Ultra-Fast Pan-Tilt Adjustment Based on Galvano Mirror

The Galvano mirror system offers precise and ultra-fast pan-tilt adjustment capability at the speed within 2 milliseconds per step. Therefore, in this study, we integrate it into our system. With the 500 fps frame-by-frame object tracking algorithm, the Galvano mirror can realize 500 Hz high-frequency visual feedback in real time.

**Figure 5:**
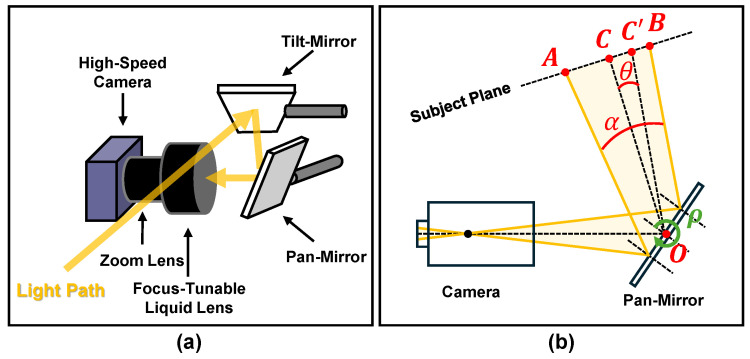
Diagram of the adjustment of the view direction: (**a**) Two Galvano mirrors are used to adjust the horizontal and vertical viewpoints. (**b**) Schematic representation of the horizontal viewpoint adjustment.

As depicted in subfigure (a) of Figure 5, the applied pan-tilt mechanism contains two Galvano mirrors. The light path is reflected twice to enter the high-speed camera. The mirror responsible for adjustment in the horizontal direction is called the pan-mirror, while the one for vertical viewpoint adjustment is called the tilt mirror.

The subfigure (b) of Figure 5 depicts the schematic representation of the horizontal viewpoint. The mirror’s angle is denoted as ρ. We can determine the relationship between the angle of the desired viewpoint adjustment Δθ, and the angle of the Galvano mirror adjustment Δρ, with the equation as follows:

(7) $$\Delta \theta =\frac{\Delta \rho }{2}.$$

Therefore, given the center of the object is $C^{\prime }$ and the current center is *C* in the image, Δρ can be calculated by Equation (8):

(8) $$\Delta \rho =2\alpha \frac{CC^{\prime }}{AB},$$

where α represents the horizontal FoV of the image, and AB refers to the width of the image in pixels. The tilt mirror, responsible for adjusting the view in the vertical direction, is adjusted in a similar method to the pan mirror and is omitted in this paper. More technical details of the pan-tilt mechanism based on the Galvano mirror can be found in the previous works discussed in Section 2.3.

### 3.4. High-Frame-Rate Frame-by-Frame Object Tracking

#### 3.4.1. Pipeline of the Proposed High-Magnification Autofocus Tracking

In this subsection, we detail the algorithms utilized for HFR frame-by-frame object tracking. These algorithms allows the accurate operation of the proposed C-AF approach based on dynamic-range focal sweep and the ultra-fast pan-tilt mechanism using Galvano mirrors.

**Figure 6:**
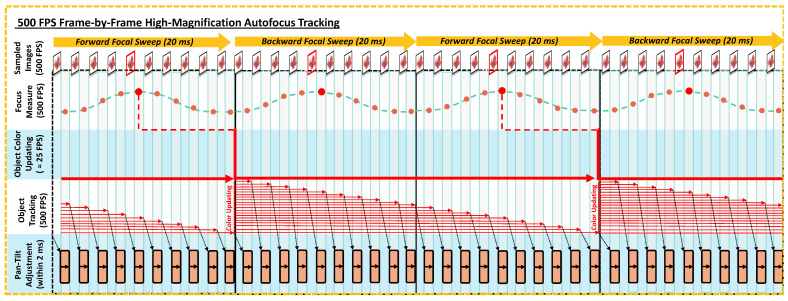
The Pipeline of the object tracking method in our system: The pipeline consists of three main threads: (1) Thread 1: 500 fps Focus Measure, (2) Thread 2: Object Main-Color Updating, and (3) Thread 3: 500 fps Frame-by-Frame Object Tracking. The focus measure algorithm is implemented to extract 50 fps well-focused images and to determine the object’s depth, adjusting the focal sweep range. The object detection algorithm operates at 25 fps, providing color updating at the same rate. Meanwhile, the object tracking algorithm achieves 500 fps frame-by-frame object tracking.

The proposed HFR object tracking pipeline is illustrated in Figure 6. It consists of three main algorithms, each running in a separate thread. These threads operate in parallel to achieve synchronization for HFR computing in real time.

The subsequent subsections cover the following contents: Section 3.4.2 outlines the algorithm for the 500 fps focus measure thread, which aims to achieve the accurate and timely adjustment of the focal sweep range and the well-focused outputs at stable 50 fps. Section 3.4.3 describes the thread for object main-color updating using the well-focused outputs from the last thread, which includes the main-color extraction algorithm based on YOLO object detection and the K-means color clustering algorithm. Section 3.4.4 explains the threads for 500 fps object tracking and pan-tilt adjustment, detailing how the main color from the second thread and all of the 500 fps images are effectively utilized for HFR frame-by-frame object tracking in real time.

#### 3.4.2. Thread 1: 500 FPS Focus Measure

This thread consists of the core algorithms for the proposed C-AF approach. We set the focus measure algorithm to run at a frequency of 50 Hz, synchronized with the focal sweep period of the liquid lens. This allows us to divide the images into groups.

In each group, 10 images, denoted as $I_{n}$, (n=1,2,…,10), are captured. During the forward focal sweep, images are captured from near to far, with $I_{1}$ captured at the minimal focus position $d_{o}^{min}$. Conversely, in the backward focal sweep, images are captured from far to near, with $I_{1}$ at the maximal focus position $d_{o}^{max}$.

We use the Canny Sharpness Detector, accelerated by a GPU, as the focus measure algorithm to obtain the edge maps, denoted as $M_{n}$, (n=1,2,…,10). The total value of the pixels in the edge map serves as the focus value, represented by $v_{n}$, (n=1,2,…,10). The focus value can be calculated by Equation (9):

(9) $$v_{n}=\sum_{x=1}^{W}\sum_{y=1}^{H}i_{M_{n}}\left(x,y\right)$$

Here, *W* and *H* represent the width and height of the edge map $M_{n}$, respectively. $i_{M}\left(x,y\right)$ refers to the pixel values in $M_{n}$.

By comparing the focus values of images captured during the focal sweep, we can extract the image with the highest focus value as the output. Thus, by running the focal sweep at 50 Hz, our method produces 50 fps of well-focused images.

In addition, as described in Section 3.2.2, we employ the DFF technique during each forward focal sweep to obtain the object’s depth information $d_{p}$ at 25 Hz. Using the latest $d_{p}$, we can immediately update the minimal and maximal focus positions after each forward focal sweep, allowing timely focal sweep range adjustment. The detailed flowchart of this thread is depicted in Algorithm 1.
**Algorithm 1:** The Proposed C-AF with 500 FPS Focus Measure.
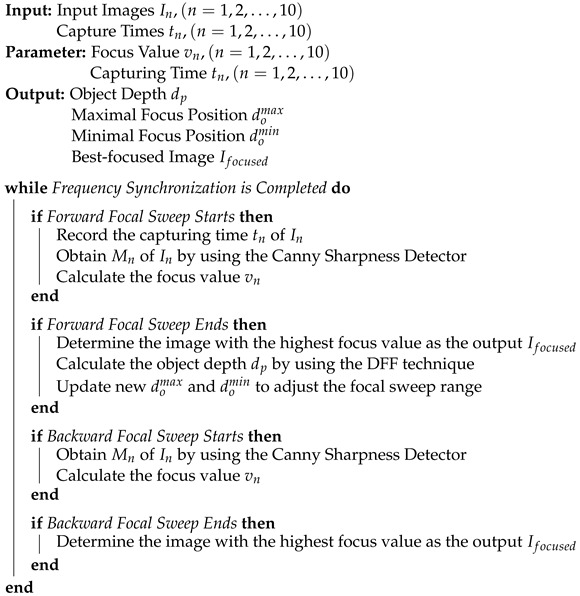


#### 3.4.3. Thread 2: Object Main-Color Updating

The novelty of our object tracking algorithm lies in the object main-color updating, which is realized by the combination of YOLO-based object detection and color-based object tracking. YOLO-based object detection offers high accuracy but incurs high computational costs, posing challenges for high-frame-rate tracking in real time. In contrast, color-based tracking offers the simplicity of the algorithm but may lack robustness for long-term tracking due to variations in real-world environments. To address this, we integrate these two methods by updating the main-color using the K-means clustering algorithm, achieving HFR frame-by-frame tracking performance.

Initially, the output $I_{\mathrm{focused}}$ from Thread 1 is processed by the YOLO algorithm to obtain the object’s region of interest (ROI), denoted as $ROI_{yolo}$, which can be described as follows:

(10) $$ROI_{yolo}=\left(x_{o},y_{o},W_{ROI},H_{ROI}\right),$$

where $x_{o}$ and $y_{o}$ denote the coordinates of the top-left pixel point, and $W_{ROI}$ and $H_{ROI}$ represent the width and height of $ROI_{yolo}$, respectively. The YOLO-based central position $c_{yolo}$ can be calculated using Equation (11):

(11) $$c_{yolo}=\left(x_{o}+\frac{W_{ROI}}{2},y_{o}+\frac{H_{ROI}}{2}\right).$$

Since the pixels in the columns and rows surrounding the boundary of $ROI_{yolo}$ often contain background colors, we introduce the core ROI $ROI_{core}$, which is defined as half the size of $ROI_{yolo}$ centered around $c_{yolo}$. The $ROI_{core}$ is specifically used for extracting the object’s main color and can be described as follows:

(12) $$ROI_{core}=\left(x_{o}+\frac{W_{ROI}}{4},y_{o}+\frac{H_{ROI}}{4},\frac{W_{ROI}}{2},\frac{H_{ROI}}{2}\right).$$

On the other hand, we create an array containing the pixels at the boundary column and row of $ROI_{yolo}$, specifically for extracting the background’s main color. This array, denoted as *P*, can be represented by Equation (13):

(13) $$P=\left[\begin{array}{cccc} a_{1,1} & a_{1,2} & \cdots & a_{1,W}\\ a_{2,1} & a_{3,1} & \cdots & a_{H-1,1}\\ a_{2,W} & a_{3,W} & \cdots & a_{H-1,W-1}\\ a_{H,1} & a_{H,2} & \cdots & a_{H,W} \end{array}\right],$$

where $a_{i,j},\left(0\leq i\leq W,0\leq j\leq H\right)$ refers to the value of the pixels at the boundary column and row of $ROI_{yolo}$.

We use the K-means clustering algorithm on the core ROI $ROI_{core}$ and the background pixels’ array *P*, resulting in the array of the object’s main colors *Q* and the array of the background’s main colors *R*, respectively. Here, we manually set the number of clusters to 4 and 2, respectively. Hence, *Q* and *R* can be described as follows:

(14) $$Q=\left[\begin{array}{cccc} q_{1} & q_{2} & q_{3} & q_{4} \end{array}\right],R=\left[\begin{array}{cc} r_{1} & r_{2} \end{array}\right].$$

The differences between the colors in *Q* and the colors in *R* can be calculated using Equation (15):

(15) $$\Delta_{i}=\frac{|q_{i}-r_{1}\left|+\right|q_{i}-r_{2}|}{2},\;\left(i=1,2,3,4\right).$$

Subsequently, the object’s main color $q_{m}$ can be determined as the color whose pixel value is the most distinguished from the pixel values of the background colors. This can be expressed as follows:

(16) $$q_{m}=q_{i}\;\mathrm{for}\;i=\underset{i\in \{1,2,3,4\}}{\arg\max}\Delta_{i}.$$

Moreover, given the main-color $q_{m}$ and setting proper maximal and minimal color-filtering thresholds $\phi_{max}$ and $\phi_{min}$, the gravity position of the pixels whose values are within the threshold range can be obtained. This gravity position is defined as the central position based on color-filtering, denoted as $c_{color}$, which can be calculated by Equation (17),

(17) $$c_{color}\left(x_{c},y_{c}\right)=\left(\frac{\sum_{(x,y)\in S}x}{n_{S}},\frac{\sum_{(x,y)\in S}y}{n_{S}}\right),$$

where *S* is the set of all pixels (x,y) whose values I(x,y) are within the range $\left[\phi_{min},\phi_{max}\right]$, which can be described as Equation (18), and $n_{S}$ refers to the total number of the pixels in the set *S*.

(18) $$S=\{\left(x,y\right)|\phi_{min}\leq I\left(x,y\right)\leq \phi_{min}\}.$$

Since the main-color of an object is often not evenly distributed over the object, there is often some error between $c_{color}$ and $c_{yolo}$. This error distance is denoted as *d*, which can be computed by Equation (19),

(19) $$d=c_{yolo}-c_{color}.$$

In our work, the distance *d* is assumed to remain constant over a short term (e.g., 40 ms in our study), we utilize *d* in the Thread 3 to determine the central position of the object in the subsequent images. The detailed steps of Thread 2 is depicted in Algorithm 2.
**Algorithm 2:** Main Color Updating and Error Distance Calculation.**Input**: Well-focused Image $I_{focused}$**Parameter:** Color-filtering Thresholds $\phi_{max}$ and $\phi_{min}$**Output**: Main color $q_{m}$               Error Distance *d*               YOLO-based ROI $ROI_{yolo}$**Step 1: YOLO Detection**Run YOLO on image $I_{focused}$ to obtain $ROI_{yolo}$Calculate central position based on YOLO $c_{yolo}$**Step 2: Main Color Obtaining**Calculate the core ROI $ROI_{core}$Obtain background pixels’ array *P*Run k-means to obtain object’s main colors *Q* and background’s main colors *R*Determine the object’s main-color $q_{m}$**Step 3: Color Filtering**Convert $q_{m}$ and pixels in $ROI_{yolo}$ to HSV formatCalculate the central position based on color filtering $c_{color}$**Step 4: Error Distance Calculation**Calculate Error distance *d*

#### 3.4.4. Thread 3: 500 fps Frame-by-Frame Object Tracking

In this thread, we utilize the YOLO-based ROI $ROI_{yolo}$, the object’s main color $c_{color}$, and the error distance *d* as inputs to achieve 500 fps frame-by-frame object tracking by using the method of color-filtering.

At first, we introduce a color-filtered ROI, denoted as $ROI_{filter}$, with additional expansion columns represented by *k*. This expansion ensures that the entire object remains within the ROI during movements while minimizing background color interference. The formulation for $ROI_{filter}$ is expressed in Equation (20).

(20) $$ROI_{filter}=\left(x_{o}-k,y_{o}-k,W_{ROI}+2k,H_{ROI}+2k\right),$$

where *k* dynamically adjusts based on the dimensions of the ROI. In practice, we assign values of 20, 40, 60, and 80 to *k* when the longest side of the ROI falls within the ranges of (400, 300), (300, 200), (200, 100), and (100, 1), respectively.

Until the next update of the inputs, we use the latest inputs to color-filter the pixel values within $ROI_{filter}$ of the subsequent images. The specific operation is the same as that of Equation (17). Moreover, the setting of the color-filtering thresholds, i.e., $\phi_{max}$ and $\phi_{min}$ in Equation (17), is also consistently the same as the setting in the last thread. Here, the central position based on color-filtering of the latest subsequent images is denoted as $c_{color}^{\prime }$.

Then, we can correct the error in $c_{color}^{\prime }$ by using the *d* calculated earlier, which can be expressed as Equation (21),

(21) $$c_{object}=c_{color}^{\prime }+d,$$

where $c_{object}$ refers to the object position in the current image. And finally, through Equation (8), the Galvano mirror’s angles can be calculated successfully. The details of this thread are depicted in Algorithm 3.
**Algorithm 3:** HFR Object Tracking based on Color Filtering.**Input**: 500 fps Image $I_{n}$            YOLO-based ROI $ROI_{yolo}$            Object’s Main-Color $q_{m}$            Error Distance *d***Parameter:** Additional Expansion Columns *k***Output**: Pan and Tilt Angles $\theta_{pan}$ and $\theta_{tilt}$**Step 1: Object Central Position Calculation with Color Filtering**Calculate $ROI_{filter}$ by expanding $ROI_{yolo}$ with *k*Convert pixels in $ROI_{filter}$ to HSV formatCalculate the central position $c_{color}^{\prime }$Obtain the object center $c_{object}$ by Equation (21)**Step 2: Pan-tilt Control**Calculate the pan and tilt angles $\theta_{pan}$ and $\theta_{tilt}$

## 4. System Configuration

**Figure 7:**
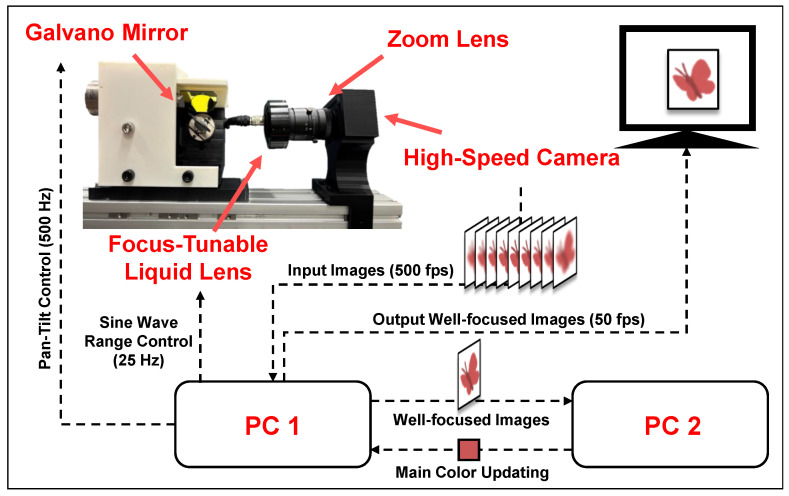
System configuration of proposed high-magnification autofocus tracking system.

The configuration of the proposed system is shown in Figure 7. The proposed system consists of a liquid lens (EL-16-40-TC VIS-20D-1-C, Optotune (Dietikon, Switzerland)) controlled by the Optotune Lens Driver 4i, a zoom lens (1/$2^{\prime \prime }$, 100 mm, F3.5, SV-10035V, VS Technology (Tokyo, Japan)), a high-speed camera (DFK27BUX287, ImagingSource (Bremen, Germany)), and a two-axis Galvano mirror system (6210H, Cambridge Technology (Bedford, MA, USA)). The camera captures images with 720 × 540 pixels at 500 fps. The liquid lens operates sine wave at 25 Hz. Therefore, this system provides focal sweeps at 50 Hz, and 10 images are captured during each focal sweep.

W utilize two PCs in the system. PC1 is with an Intel Core i7-9800X CPU and a NVIDIA Quadro RTX 5000 GPU, and PC 2 is with a AMD Ryzen 9 5900HX CPU and a NVIDIA GeForce RTX 3080 Laptop GPU. PC 1 extracts well-focused images and controls the liquid lens and the Galvano mirror. PC 2 handles object detection tasks. Thus, PC 1 sends well-focused images to PC 2, where YOLO-based object detection and main-color extraction are implemented. PC 2 sends the results to PC 1. The data transmission is facilitated via TCP. The details of the computation of the core algorithms implemented in the pipeline are recorded in Table 3.

## 5. Experiments

In this section, we present four experiments to demonstrate the capabilities of our system. Experiment 1 evaluates the C-AF performance with three small, fast-moving targets, highlighting the effectiveness of the proposed C-AF approach. Experiment 2 quantifiedly assesses the depth measurement accuracy and robustness of the C-AF method under varying lighting conditions and depths. Experiment 3 demonstrates the feasibility of the proposed object tracking method, showcasing that all the 500 fps images, including both well-focused and less-focused images, can be utilized effectively. Finally, Experiment 4 provides a comprehensive assessment by tracking a fast-moving, small target to illustrate the performance of HFR high-magnification C-AF tracking.

### 5.1. Experiment 1: Continuous Autofocus Based on Dynamic-Range Focal Sweep

This experiment demonstrates the C-AF performance by analyzing the depth measurement results and the object detection’s success rates on the well-focused images. In this experiment, we used three small, fast-moving objects. Their information is listed in Table 4.

**Figure 8:**
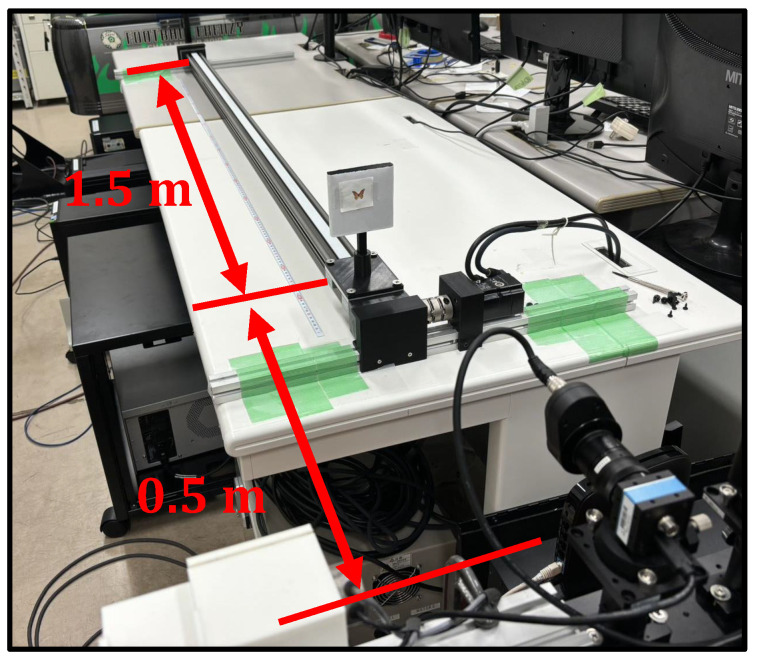
The environment of Experiment 1.

As depicted in Figure 8, a linear actuator was positioned in front of the camera, enabling the platform to move back and forth within a depth range of 0.5 m to 2.0 m. In our experiment, objects were placed on the linear actuator’s movable platform and moved back and forth at speeds of 3 m/s.

**Figure 9:**
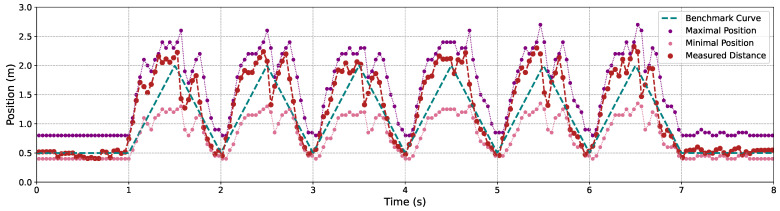
The focal sweep adjustment and the depth measurement results using the proposed C-AF with butterfly model’s movements.

**Figure 10:**
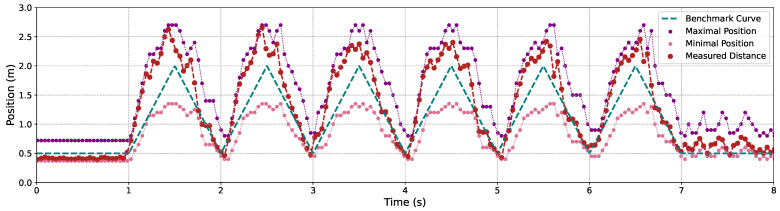
The focal sweep adjustment and the depth measurement results using the proposed C-AF with qr code’s movements.

**Figure 11:**
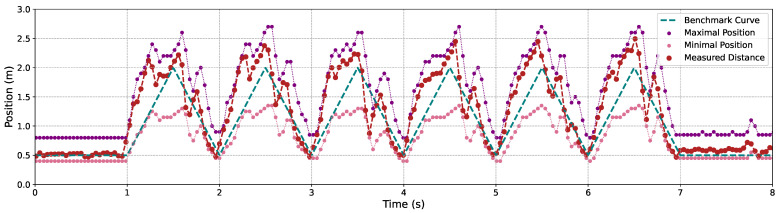
The focal sweep adjustment and the depth measurement results using the proposed C-AF with screw’s movements.

Figure 9, Figure 10 and Figure 11 illustrate the variation in the maximal and minimal positions, as well as the depth distance measured by our proposed C-AF approach, alongside the benchmark depth distances for Objects 1, 2, and 3, each operating under 3 m/s back-and-forth movements. It is evident that measurement accuracy improves when objects are closer to the camera compared to when they are at a greater distance. This is attributed to the smaller gaps between adjacent images, which fit the shallower DoF at closer distances. Conversely, at greater distances, the DoF is deeper, causing the focal sweep range to widen, which leads to less precise measurement results.

Therefore, despite the increased measurement errors between the measured and benchmark values at greater distances, our method effectively and promptly adjusts the focal sweep ranges. Consequently, the objects remain within the focal sweep range, allowing us to continuously obtain well-focused images at stable frame rates.

**Figure 12:**
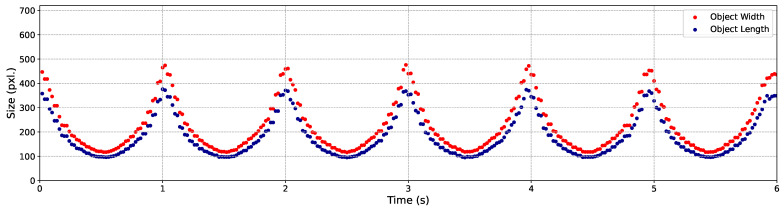
Variation of size with butterfly model’s movements.

**Figure 13:**
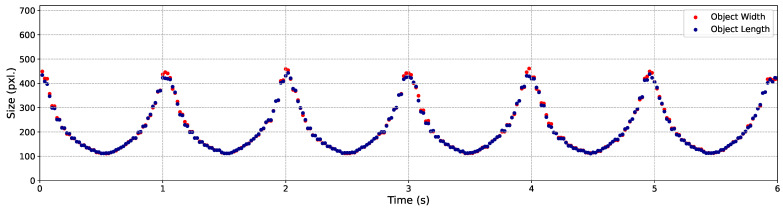
Variation of size with QR code’s movements.

**Figure 14:**
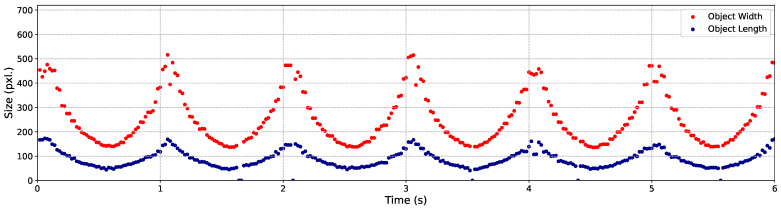
Variation of size with screw’s movements.

In the next step of this experiment, we evaluated the C-AF performance by employing YOLO to determine whether the objects in the well-focused images, captured during the 3 m/s back-and-forth movements, could be successfully detected. The YOLO results are visualized by showing the variation in the sizes of the three objects during the 3 m/s back-and-forth movements, as illustrated in Figure 12, Figure 13 and Figure 14, respectively.

**Figure 15:**
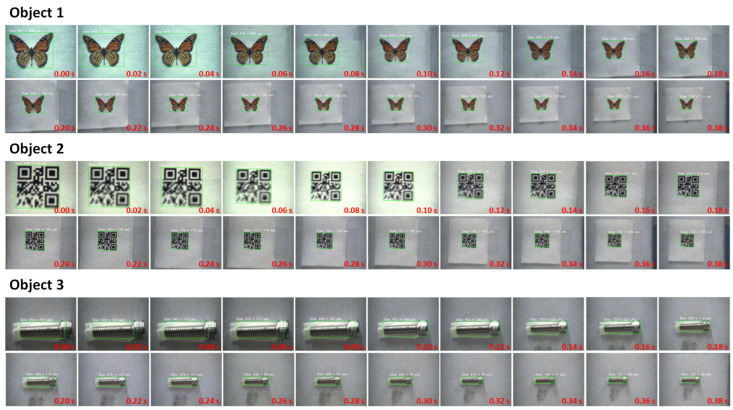
Output images’ detection results with object movements at 3 m/s.

In these figures, the width (the pixel amount in the horizontal orientation) and length (the pixel amount in the vertical orientation) of the objects, which are also the width and the height of the bounding boxes, are recorded and represented by points in red and blue, respectively. It is evident that the majority of the output images were correctly detected. Specifically, within 6 s of back-and-forth movements, a total of 300 frames of well-focused images were captured. The detection success rates for Objects 1, 2, and 3 were 100%, 100%, and 98.33%, respectively. The output images obtained within the first 0.38 s are showcased in Figure 15.

Initially, the objects were positioned at the closest depth, where the DoF was narrowest, posing the greatest challenge for achieving C-AF. Similarly, for our system, this closest distance is the most challenging compared to relatively farther distances, where the DoF is not as narrow. This is because the focal sweep range had to be relatively small to fit the extremely narrow DoF, placing high demands on our system to adjust the focal sweep range correctly and timely so that the moving object remained within the range.

As shown in the subfigures of Figure 15, as the objects moved farther away from 0.00 s to 0.38 s, the objects in the output images were sufficiently sharp and successfully detected. This demonstrates the system’s capability to maintain focus and detect objects effectively, even as the distance and DoF conditions change rapidly.

However, some unsatisfactory results were observed for Object 2. This can be attributed to two primary reasons:

- **Over-Narrow DoF:** When the focused subject plane was too close, the DoF became excessively narrow, possibly causing only part of Object 2 to be in focus. To ensure that the entire object is focused in the output, we believe that our previous work on a real-time DoF extension algorithm can be utilized effectively [48].
- **Insufficiently Sensitive Focus Measure Algorithm:** The strength of the edge features of the QR code is too homogeneous, making it difficult to distinguish the best-focused image precisely, unlike Object 1 and Object 3, where edges with different strengths are contained. To address this issue, we plan to use a more sensitive focus measure algorithm in future iterations.

In summary, this experiment showcases the impressive performance of the C-AF approach based on dynamic-range focal sweep. As proved by the results, the proposed C-AF approach consistently provides well-focused images at a stable 50 fps, even when objects operate in rapid movements in the depth direction. While there is room for improvement in handling specific challenges, the results indicate that our proposed C-AF approach is highly effective in most scenarios.

### 5.2. Experiment 2: Effectiveness and Robustness Analysis of C-AF Based on Dynamic-Range Focal Sweep

In this experiment, we established three distinct lighting scenarios to showcase the effectiveness and robustness of depth measurement employing the proposed C-AF. The lighting conditions, in the order from bright to dark, are listed as follows:

- **Lighting Condition (i):** Fluorescent Lamps + LED Light Illuminator;
- **Lighting Condition (ii):** Indoor natural lighting with fluorescent lamps;
- **Lighting Condition (iii):** Indoor natural lighting.

We set Object 1, 2, and 3, which we have utilized in Experiment 1, at 0.6 m, 0.9 m, 1.2 m, and 1.8 m, respectively. Then, our system was operated the proposed C-AF approach to obtain the well-focused images continuously.

To quantify the effectiveness and robustness, we defined the depth measurement absolute error (DMAE) and the focus value relative loss (FVRL). DMAE calculates the averaging deep measurement error with the measured depth values and the true value. FVRL calculates the percentage loss of the focus values of the well-focused outputs compared to the focus value of the image whose focus is at the optimal focus position. The formulas of DMAE and FVRL are expressed in Equations (22) and (23),

(22) $$DMAE=\frac{1}{N}\sum_{k=1}^{N}\left|d-{\hat{d}}_{k}\right|,$$

(23) $$FVRL=\frac{1}{N}\sum_{k=1}^{N}\left|\frac{v-{\hat{v}}_{k}}{v}\right|\cdot 100\%,$$

where *d* represents the true value of the object depth, $\hat{d}$ refers to the measured depth, *v* denotes the focus value of the image at the optimal focus, and $\hat{v}$ represents the calculated value of the focus value. In the experiment, the time *N* was set to 500.

The experimental results of the DMAE are depicted in Table 5. It can be found that the proposed C-AF can correctly measure the object depths. Among them, the lowest error is 0.026 m, appearing when objects were set at 0.6 m at the brightest lighting condition, while the largest error is at 0.520 m, where the objects were at a distance of 2.1 m at the darkest lighting condition. The reasons for this difference are as follows: Firstly, because the DoF is narrower when the object’s depth is closer to the camera, the focal sweep range that we manually set was smaller. Thus, the gap between the adjacent images was closer, leading to precise results. Secondly, with brighter lighting conditions, the edge information on the objects becomes more outstanding. This causes a more distinguished focus value of the well-focused image compared to the less-focused ones. Moreover, the C-AF task can be operated with all objects at all distances and light conditions in this experiment, showcasing its good robustness in various environments.

The results of the FVRL are shown in Table 6. Similar to the former results, the best result in this table appears when the object is at 0.6 m at the brightness lighting condition. However, we can find that the FVRL is less related to the lighting conditions and more related to the distance of the object. This is because when objects are at a longer distance, there are fewer pixels that contain edge information. Thus, when the image is not at optimal focus, the percent loss is high. While this suggests that there is room for improvement in the accuracy of the proposed C-AF approach for objects at far distances, it can be gleaned from the results of Experiment 1 that the recognition rate for objects at a distance was not affected. Moreover, it can be found that the type of object profoundly affects the FVRL. Among the three objects used in the experiment, the QR Code always has the best performance, while the screw is always the worst. This indicates that the performance of the proposed C-AF is affected by the slight edge information of the object. If the object contains many slight edges, it is easy to affect the final result. Therefore, in our future research, we will try to shorten the focal sweep range in order for the focus position of the well-focused image can be closer to the optimal focus as much as possible.

### 5.3. Experiment 3: Feasibility Demonstration of the Proposed HFR Object Tracking Method

In this experiment, we positioned objects used in Experiment 1 at distances of 0.75 m, 1.25 m, and 1.75 m, respectively, while implementing the proposed C-AF approach. Thus, during a focal sweep, 10 images with various focuses around the optimal focus position of each object were captured. We applied the proposed HFR object tracking method to calculate the object position in all of the images captured during the focal sweep. Because the object is positioned at a fixed position, the center position obtained by YOLO on the frame with the highest focus value can be considered as the benchmark of the object’s position, and the displacement between the central position through the tracking algorithm based on the color-filtering is defined as the object tracking error in the unit of pixels.

**Figure 16:**
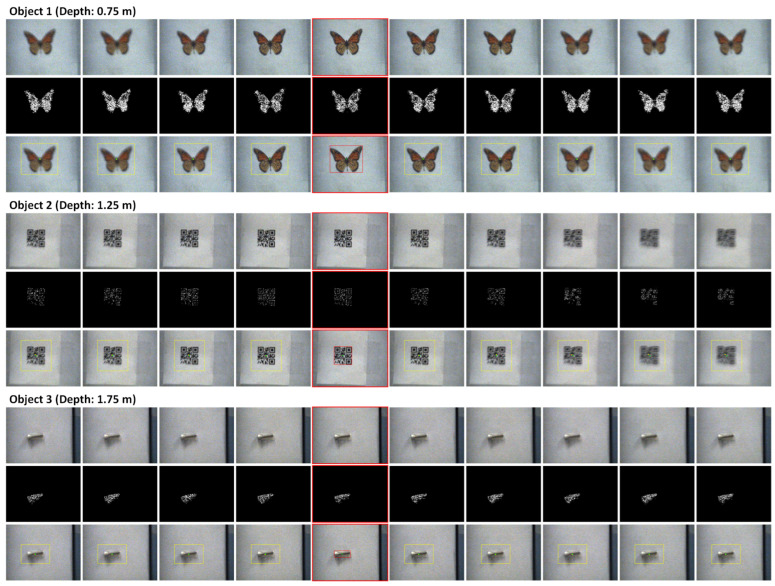
Results of proposed HFR object tracking method for multiple objects at different distances: These figures were captured at 500 fps sequentially during one process of the focal sweep. The first, the second, the third columns shows the original images, color-filtered maps, and the results.

The original images, color-filtered maps, and tracking results for Object 1, Object 2, and Object 3 at depth distances of 0.75, 1.25, and 1.75 are displayed in Figure 16. The first column of each object showcases the original images captured in chronological order by the high-speed camera during an entire period of focal sweep. It can be seen that the images vary from blurry to sharp and then back to blurry during the focal sweep, as it captures images within a short range around the optimal focus. By comparing the focus values of each image using the implemented focus measure algorithm, the best-focused images for Object 1, Object 2, and Object 3 were found in the fifth frame. These images, along with their color-filtered maps and YOLO-based results, are highlighted with red boxes. The green points in the YOLO-based results represent the central positions obtained by YOLO. The second column for each object displays the color-filtered maps. It is easy to find that blurriness in less-focused images is slight; thus, the color information of the objects in these images is still reliable to be used for object tracking. Subsequently, as shown in the third column for each object, our tracking algorithm is employed on the remaining images, with central positions and color-filtered areas calculated through the color-filtering process indicated in yellow. As shown, the tracking error is very small because the distances between the yellow and green points are only a couple of pixels.

Since the objects were stationary, the distance between the YOLO-detected result of the best-focused image and the tracking algorithm’s results of the other images can be considered as the object tracking error. Table 7 quantified this error with Object 1, 2, and 3, at the distances of 0.75 m, 1.25 m, and 1.75 m, respectively.

In summary, this experiment assessed the viability of the proposed HFR object tracking method due to the small object tracking errors. The results affirm two key points: firstly, because the color information of the object has sufficient robustness against the slightly defocused blur, accurate and high-speed object tracking in the short term is achievable by using the color information and the object position data given by the image captured previously; secondly, despite containing less-focused images, the blur of them are sufficiently slight to be utilized effectively in the proposed HFR object tracking algorithm.

### 5.4. Experiment 4: HFR Frame-by-Frame High-Magnification Autofocus Tracking

**Figure 17:**
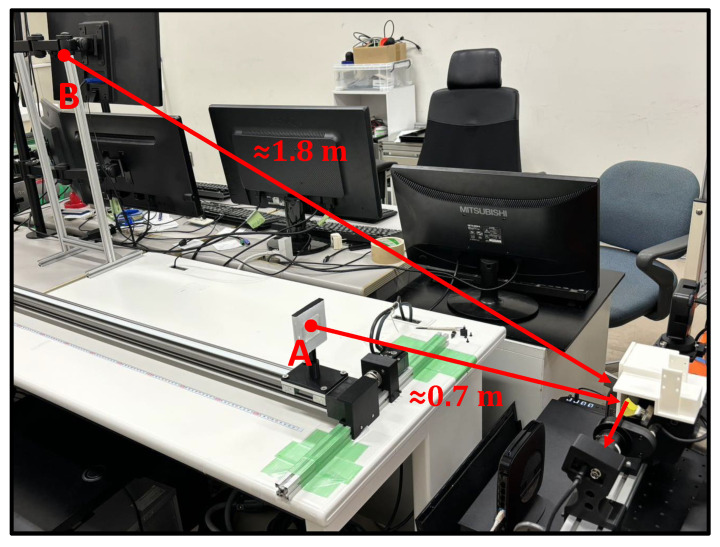
Environment of Experiment 4.

This experiment showcases the swift high-magnification tracking ability of our proposed AVS. We held the butterfly model target and moved it back-and-forth rapidly between Position A (depth ≈ 0.7 m) and Position B (depth ≈ 1.8 m) 3 times. The experimental environment is depicted in Figure 17.

**Figure 18:**
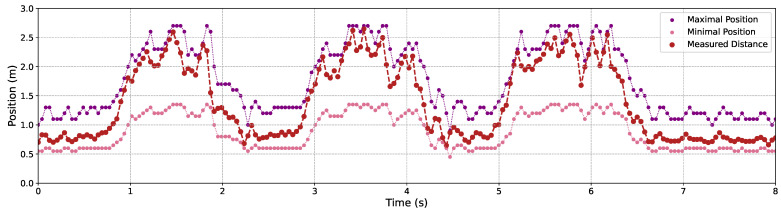
The focal sweep adjustment and the depth measurement results using the proposed C-AF with the object’s movements.

We recorded the measured depth distances of the object and the maximal and minimal positions of the dynamic-range focal sweep throughout this process, as shown in Figure 18. Similar to the relevant results of Experiment 1, it can be found that the object’s depth can be measured correctly and the adjustment of the focal sweep range was timely so that the object consistently remained within the focal sweep range. It is a prerequisite evidence to suggest that well-focused images of the object can be obtained continuously throughout its whole movement.

**Figure 19:**
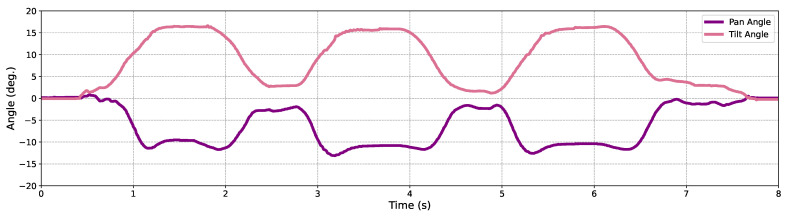
Viewpoint’s variation during the object movement.

In addition, in order to demonstrate the effectiveness of the proposed HFR high-magnification object tracking pipeline, along with the ultra-fast pan-tilt mechanism based on the Galvano mirror, we also recorded the pan-tilt angle of the viewpoint, as illustrated in Figure 19. As observed from the trends of these curves, the fast-moving small object was tracked steadily and continuously.

**Figure 20:**
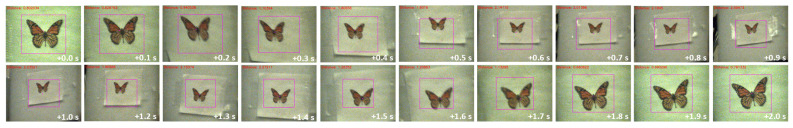
Some results of HFR high-magnification object tracking.

Figure 20 showcases some output images obtained during the object’s movements. Here, this figure displays the output images at 0.1-s intervals. From the first image (time: +0.0 s) to the twentieth image (time: +2.0 s), the object moved from Position A to B and then back to Position A, staying in focus throughout. The purple box in each photo indicates the color-filtered ROI, and it is clear that the object remained within this area during the movement. Additionally, purple dots denote the computed object center position. It’s important to note that some small latency appears in these results because in our multi-threading program, the displayed images were the latest ones, while the results drawn on the current images were obtained from their previous frames.

In summary, the results of this experiment emphasize the exceptional tracking performance of our system. For most previous tracking systems, tracking small moving targets with high magnification is inherently difficult due to the narrow DoF and small FoV. However, our proposed C-AF approach based on dynamic-range focal sweep coupled with the HFR frame-by-frame object tracking algorithm, which is the main contribution of our work, adeptly overcomes this challenge.

## 6. Conclusions

In this paper, we propose a novel high-magnification tracking system. This system integrates an innovative C-AF approach enabled by a high-speed camera, a focus-tunable liquid lens, and a new HFR frame-by-frame object tracking pipeline with the ultra-fast pan-tilt mechanism based on the Galvano mirror. The proposed C-AF approach promotes the AF method based on the focal sweep by introducing the DFF technique.

Specifically, in order to accelerate the C-AF, we propose a new C-AF approach based on dynamic-range focal sweep, taking full use of the high adjustment capability of the liquid lens. This new method effectively minimizes the frame numbers used in the focal sweep, providing well-focused images at stable and higher frame rates. In our work, the high-speed camera captures at 500 fps, and the liquid lens was set to perform focal sweeps at 50 Hz, resulting in stable and continuous 50 fps well-focused output image sequences. And more importantly, because the remaining images are only slightly less focused, all 500 fps images can be utilized for object tracking.

Moreover, the proposed HFR frame-by-frame object tracking pipeline hybridizes the deep-learning-based method and the feature-based method, specially designed to meet the demand of the high-magnification tracking on the high-frequency visual feedback owing to the narrow FoV. It utilizes YOLO to obtain object detection results, and by using the K-means clustering algorithm, the object’s main color can be updated. With the consistently updated color information, the proposed system achieves advanced 500 fps frame-by-frame high-magnification object tracking based on color filtering in real time.

In this paper, we conducted a full experiment to demonstrate the capability of our proposed system. Experiment 1 and Experiment 2 were conducted using multiple objects positioned at a wide range of depths and different lighting conditions to fully analyze the effectiveness and the robustness of the proposed C-AF approach. Experiment 3 analyzes the accuracy of the HFR object tracking algorithm by quantifying the tracking error. Finally, Experiment 4 showcases the advanced performance of our system for high-magnification tracking.

However, our system has some limitations, including potential poor performance with objects lacking sufficient edge information and reduced robustness in scenes with varying light source intensities. Additionally, color-based object tracking may suffer from over-reliance on a single object feature. Future work will focus on exploring alternative algorithms to enhance the stability and robustness of the system.

## Figures and Tables

**Table 1:** Comparison of the proposed C-AF approach with previous methods.

Method	Necessity of Additional Sensors	Capability of Depth Measurement	Frame Rate of Well-Focused Images
**(i) Contrast-Based AF [37]**	**No**	No	Not Stable
**(ii) Phase-Based AF [20,35,36]**	Yes	No	Not Stable
**(iii) Active-Based AF [22,23,33,34]**	Yes	**Yes**	**Stable**
**(iv) AF based on Global-Range Focal Sweep [26,27]**	**No**	**Yes**	Stable but Relatively Low
**(v) AF based on Dynamic-Range Focal Sweep (This Study)**	**No**	**Yes**	**Stable and Relatively High**

**Table 2:** Comparison of the proposed system with previous AVSs.

Active Vision System	Speed of Pan-Tilt Adjustment	Applicability to High-Magnification Tracking in Pan-Tilt Directions	Applicability to High-Magnification Tracking in Depth Direction
**(i) Camera-Rotation-Based** **Fixed Focus AVS [66]**	Slow. (Pan: 170 deg./s, Tilt: 77 deg./ms)	Only for Low-Speed Objects	No
**(ii) Camera-Rotation-Based** ** Autofocus AVS [67]**	Slow (Not Explicitly Stated in the Paper)	Only for Low-Speed Objects	Only for Low-Speed Objects
**(iii) Galvano-Mirror-Based** **Fixed Focus AVS [68]**	**Fast** (Pan/Tilt: 5.7 deg./ms, Frequency: 500 Hz)	**Yes**	No
**(iv) Galvano-Mirror-Based** **AVS with Autofocus Based** **on Global-Range Focal** **Sweep [27]**	Fast But Low Feedback Frequency (Pan/Tilt: 10 deg./ms, Frequency: 10–15 Hz)	Only for Low-Speed Objects	**Yes**
**(v) Galvano-Mirror-Based** **AVS with Autofocus Based** **on Dynamic-Range Focal** **Sweep (This Study)**	**Fast** (Pan/Tilt: 10 deg./ms, Frequency: 500 Hz)	**Yes**	**Yes**

**Table 3:** Details of the computation of the core algorithms implemented in the pipeline.

Algorithm Item	The Utilized PC	Cost Time per Image (Unit: ms)
**Focus Measure Algorithm**	PC 1	1.7
**YOLOv8 Detection**	PC 2	11.8
**K-means Clustering**	PC 2	27.0
**Tracking Based on Color-Filtering**	PC 1	1.8

**Table 4:** The Object tracking error with different objects at different distances.

Object Item	Width (cm)	Length (cm)
**Butterfly (Object 1)**	1.6	1.2
**QR Code (Object 2)**	1.5	1.5
**Screw (Object 3)**	1.9	0.7

**Table 5:** Depth Measurement absolute error at different depth distances in different lighting condition (unit: m).

Object	Lighting Condition (i)						Lighting Condition (ii)						Lighting Condition (iii)					
	0.6 m	0.9 m	1.2 m	1.5 m	1.8 m	2.1 m	0.6 m	0.9 m	1.2 m	1.5 m	1.8 m	2.1 m	0.6 m	0.9 m	1.2 m	1.5 m	1.8 m	2.1 m
**1.**	0.024	0.082	0.131	0.191	0.156	0.307	0.021	0.172	0.132	0.165	0.181	0.207	0.064	0.054	0.155	0.226	0.318	0.582
**2.**	0.035	0.081	0.159	0.184	0.170	0.239	0.051	0.089	0.180	0.192	0.149	0.296	0.039	0.077	0.160	0.140	0.327	0.454
**3.**	0.020	0.070	0.181	0.160	0.186	0.215	0.054	0.119	0.186	0.213	0.179	0.510	0.079	0.117	0.167	0.299	0.199	0.524
**Avg.**	0.026	0.078	0.157	0.178	0.171	0.254	0.044	0.127	0.166	0.190	0.170	0.338	0.061	0.083	0.161	0.222	0.281	0.520

**Table 6:** Focus value relative loss at different depth distances in different lighting conditions.

Object	Lighting Condition (i)						Lighting Condition (ii)						Lighting Condition (iii)					
	0.6 m	0.9 m	1.2 m	1.5 m	1.8 m	2.1 m	0.6 m	0.9 m	1.2 m	1.5 m	1.8 m	2.1 m	0.6 m	0.9 m	1.2 m	1.5 m	1.8 m	2.1 m
**1.**	6.50%	12.56%	23.21%	17.03%	15.42%	31.54%	6.08%	20.73%	25.50%	21.32%	24.46%	31.03%	35.36%	17.98%	23.48%	20.18%	20.67%	20.66%
**2.**	2.01%	2.58%	2.82%	2.44%	14.66%	25.49%	3.97%	3.71%	3.86%	8.53%	12.02%	18.10%	1.87%	2.15%	3.71%	5.06%	16.77%	11.70%
**3.**	9.01%	18.02%	23.38%	44.98%	48.67%	35.88%	18.05%	31.33%	23.95%	30.50%	42.57%	38.50%	8.56%	17.56%	28.19%	49.9%	32.32%	28.93%
**Avg.**	5.84%	11.05%	16.47%	21.48%	25.26%	30.97%	9.37%	18.59%	17.77%	20.12%	26.68%	29.21%	15.26%	12.56%	18.46%	25.05%	23.25%	20.43%

**Table 7:** The object tracking error with different objects at different distances (unit: pxl.).

Object Item	0.75 m	1.25 m	1.75 m
**Butterfly (Object 1)**	4.93	0.89	2.66
**QR Code (Object 2)**	14.81	12.23	3.11
**Screw (Object 3)**	2.72	3.36	3.83
**Avg.**	7.49	5.49	3.20

## Data Availability

The data are not publicly available due to it is obtained using our self-developed system and is not universal.
